# The A818–6 system as an in-vitro model for studying the role of the transportome in pancreatic cancer

**DOI:** 10.1186/s12885-020-06773-w

**Published:** 2020-03-30

**Authors:** Doaa Tawfik, Angela Zaccagnino, Alexander Bernt, Monika Szczepanowski, Wolfram Klapper, Albrecht Schwab, Holger Kalthoff, Anna Trauzold

**Affiliations:** 1 Institute for Experimental Cancer Research, Christian-Albrechts-University of Kiel, Arnold-Heller Str. 3, 24105 Kiel, Germany; 2 Clinic for Internal Medicine II, Christian-Albrechts-University of Kiel, UKSH, Kiel, Germany; 3 Institute of Pathology, Hematopathology Section and Lymph Node Registry, Christian-Albrechts-University of Kiel, UKSH, Kiel, Germany; 4 Institute of Physiology II, Westfälische Wilhelms-Universität, Münster, Germany

**Keywords:** PDAC, Transportome, Ion channels, Differentiation, Malignant transformation, 3D culture, Hollow spheres, Microarray

## Abstract

**Background:**

The human pancreatic cancer cell line A818–6 can be grown in vitro either as a highly malignant, undifferentiated monolayer (ML) or as three-dimensional (3D) single layer hollow spheres (HS) simulating a benign, highly differentiated, duct-like pancreatic epithelial structure. This characteristic allowing A818–6 cells to switch from one phenotype to another makes these cells a unique system to characterize the cellular and molecular modifications during differentiation on one hand and malignant transformation on the other hand. Ion channels and transport proteins (transportome) have been implicated in malignant transformation. Therefore, the current study aimed to analyse the transportome gene expression profile in the A818–6 cells growing as a monolayer or as hollow spheres.

**Methods & Results:**

The study identified the differentially expressed transportome genes in both cellular states of A818–6 using Agilent and Nanostring arrays and some targets were validated via immunoblotting. Additionally, these results were compared to a tissue Affymetrix microarray analysis of pancreatic adenocarcinoma patients’ tissues. The overall transcriptional profile of the ML and HS cells confirmed the formerly described mesenchymal features of ML and epithelial nature of HS which was further verified via high expression of E-cadherin and low expression of vimentin found in HS in comparison to ML. Among the predicted features between HS and ML was the involvement of miRNA-9 in this switch. Importantly, the bioinformatics analysis also revealed substantial number (*n* = 126) of altered transportome genes. Interestingly, three genes upregulated in PDAC tissue samples (GJB2, GJB5 and SLC38A6) were found to be also upregulated in ML and 3 down-regulated transportome genes (KCNQ1, TRPV6 and SLC4A) were also reduced in ML.

**Conclusion:**

This reversible HS/ML in vitro system might help in understanding the pathophysiological impact of the transportome in the dedifferentiation process in pancreatic carcinogenesis. Furthermore, the HS/ML model represents a novel system for studying the role of the transportome during the switch from a more benign, differentiated (HS) to a highly malignant, undifferentiated (ML) phenotype.

## Background

Despite the modern advances in cancer therapy, PDAC remains a devastating disease, owing to its late and difficult diagnosis on the one hand and the aggressiveness of the PDAC cells on the other hand [1]. Even with the low incidence of PDAC, notoriously, it ranks fourth among the cancer related deaths in the United States and Europe [2, 3]. Although most of cancer related deaths are predicted to be declining by 2020, the death rate from pancreatic cancer will increase and be the second cause of cancer-related deaths within the next decades [4, 5]. Therefore, a better understanding of PDAC development is still urgently needed.

PDAC is an epithelial tumour that arises from the cells of the pancreatic duct [6] or from acinar cells undergoing acinar to ductal metaplasia (ADM) thus, exhibiting a ductal phenotype [7]. In healthy exocrine pancreas, epithelial cells align with neighbouring cells and adhere to the basement membrane to create a well organised epithelial sheet and give rise to three-dimensional tubuloacinar glands [8]. Moreover, the continuous layer of pancreatic ductal epithelial cells possess clear epithelial features including specialized cell-to-cell contacts of tight junction and a polarized morphology, by which the cells exhibit three types of surfaces. The basal surface interacts with the extracellular matrix, the subsequent lateral surfaces communicate with other cells, and the luminal surface faces the lumen [8]. Therefore, a correct three-dimensional (3D) organization and tissue architecture are core requirements for tissue homeostasis, i.e. control of cellular proliferation, survival, regulating cell adherence and differentiation [9]. Particularly in exocrine glands, the polarity of the epithelial cells is also essential for the control of the cellular absorption and secretion [8, 10].

The pancreatic ductal system secretes an enormously bicarbonate-rich fluid, which is required to neutralize the acidic chime entering the duodenum and to provide an optimal pH microenvironment for the activity of digestive enzymes [11]. From a physiological perspective, the ion/fluid transport causes a transepithelial osmotic gradient that directly influences the intracellular volume [12, 13]. The secretory cells counteract the fluctuation of cellular volume, by coordinating the net transport of potassium, sodium and chloride ions across the luminal and basolateral plasma membranes [14]. That implies that cell volume homeostasis is an essential part of the secretory function of the pancreatic ductal cells [15]. Disruption of this homeostatic state was reported in pathophysiological conditions of renal diseases or brain ischemia, causing dysregulation of cell volume regulatory transporters (imbalance of sodium and potassium intake) and an impaired acid/base transport (reviewed in Hoffmann, Lambert, & Pedersen, 2009). Moreover, the huge acid-base fluxes across the ductal epithelium require a very efficient control of the intracellular pH homeostasis [16]. Possibly, the ability of pancreatic cells to cope with such enormous acid-base fluxes also contributes to the aggressiveness of PDAC [17].

The switching of cell polarity alters the localization of the transport proteins [18]. As a result, some apical ion channels and transport proteins move to the rear end, whereas some basolateral transporter re-localize at the leading edge of the migrating tumour cells. On the one hand, that causes the dysregulation of cellular volume homeostasis, as observed in many secretory epithelia afflicted by cancers i.e. colorectal, gastric, mammary gland and pancreatic [19–22]. On the other hand, it may contribute to cell migration [23]. Therefore, a focused analysis of the transportome in differentiated/undifferentiated cells will help to define the role of ion channels and transporters in PDAC.

Hitherto, 3-dimensional (3D) culturing is not intensively investigated [24] partly because the assessment and scalability of the biological behaviour of the cells under 3D culturing condition is still problematic. However, it is generally accepted that the in vitro organotypic 3D cell culture system better resembles the physiological condition of the tissues in vivo, in regards of i) architectural organization and the processes of glandular lumen formation, ii) cell-to-cell interaction, and iii) the role of cancer genes in cell polarity, therefore, allowing the investigation of the different aspects of tumour biology and pathophysiology [25]. Among the 3D culture methods, the spontaneous cell aggregation is a widely used technique [25, 26]. Hereby, malignant cells spontaneously aggregate on the substrate preventing cell adherence and promoting the formation of spheroids that grow in suspension [25]. Beforehand, it has been described that other human pancreatic cancer cell lines (HPAF-II, HPAC and PL45) derived from PDAC [26] can develop spheroids with a compact structure similar to avascular tumours.

The human PDAC cell line A818–6 bears an activating mutation in codon 12 of the KRAS gene [(G12R); personal communication Franziska Wilhelm, Institute of Pathology, CAU Kiel], which is the most common alteration in PDAC. The A818–6 cells can be grown in two different physical forms, 2D or 3D model, and both are significantly different. When the cells are grown under 3D culturing conditions, A818–6 cells form hollow sphere (HS) structures and when grown under 2D conditions, the cells grow as a monolayer (ML). The 3D HS structure is formed when the A818–6 cells are not allowed to adhere to the bottom of the culture flask/plate. Under these conditions, of one-layer cell spheres with a hollow centre are build, hence the name hollow spheres. In contrast, when the A818–6 cells are while the ML cells were allowed to adhere, and they grow as a monolayer ML on the bottom of the culture flask/plate. It was formerly reported that the cells in HS proliferate slower than ML and display morphological and functional polarity. Furthermore, in contrast to ML cells, they are not able to form tumours when orthotopically inoculated into SCID-mice. Importantly, A818–6 cells possess a high degree of cellular plasticity. When HS are mechanically disrupted they regrow as a ML regaining all the founder ML attributes and vice versa [27–29]. This cellular plasticity enables the A818–6 cell to transform from a rather benign/differentiated cell (HS) state a fully malignant/mesenchymal (ML) state. Cellular plasticity is essential to enable cancer cells to migrate to other organs and form metastases [30]. This cell line provides an opportunity to study the role of proteins critically involved in the process of epithelial-mesenchymal transition (EMT) and the reversal of this process, MET. In the current study, a whole genome-wide analysis of the two forms of A818–6 was performed to predict how cellular plasticity governs the malignant transformation in this cell line. Specifically, we aimed to explore the possible utility of this model in studying the role of transportome in PDAC.

## Methods

### Cell culture

The cell line A818 was originally isolated from the ascites fluid of a 75-years old female patient suffering from pancreatic adenocarcinoma. A dilution series was previously performed in our Institute and clone number 6 (A818–6) that was able to form 3D hollow spheres when seeded on agar-plated wells was isolated [29]. In the present study, A818–6 cell line was cultured in RPMI Medium 1640 (Gibco, Life Technologies) with 1% Glutamax (Gibco, Life Technologies), 1% Sodium Pyruvate 100 M (Gibco, Life Technologies) and 10% foetal bovine serum (PAN, BIOTECH GmbH). The cells were incubated at 37 °C under 5% CO_2_ humid atmosphere and they were grown both as a 2D ML via direct seeding of the cells (3 × 10^5^/ml) in 6-well culture plates or as HS. To create hollow spheres, cells were seeded at a density of 1 × 10^5^ per well in 3 mL media on a culture plate pre-coated with 3.1% agarose (6-well format). Following HS formation, which took 8–10 days, the HS were transferred to normal cell culture plates (6-well format) for further maturation.

### Immunoblotting analysis

Whole cell lysates were prepared using RIPA buffer and analysed via immunoblotting. Antibodies were purchased from: Santa Cruz Biotechnology, Inc., Heidelberg, Germany (anti-Vimentin, anti-LDHA and anti-LDHB); BD transduction laboratories, Heidelberg, Germany (ant-E-cadherin); Sigma Aldrich, Taufkirchen, Germany (anti-β-actin), Cell Signalling Technologies; Frankfurt am Main, Germany (anti-c-myc, anti-HMGA2, anti-p27 and HRP-conjugated anti-mouse and anti-rabbit).

### Microarray

The cell microarray experiment was performed to analyse the changes in gene expression in A818–6 cells grown as a ML or as HS. RNA was extracted using Qiagen RNeasy mini kit (Qiagen, Germany). The amount and purity of RNA was measured by Nanodrop (Thermo Scientific) and the Agilent 2100 bioanalyser system. The experiment was performed using the Agilent technology Sureprint G3 Human GE 8 × 60 K (Agilent, Santa Clara, CA, USA) and it was analysed by imaGenes (Agilent Expression Profiling service, Berlin). Regarding the tissue microarray, the mRNA expression levels were investigated using U133 A/B Affymetrix GeneChip, the detailed methodology was previously reported [31, 32]. The patients gave consents and the ethical committee approved the original study [31], which performed a whole genome expression analysis from which we have only taken a subset for further interpretation. The ethical committee of medical faculty of Christian Albrechts university of Kiel approved the study under the number A110/99. The gene expression database used in this study including the patients’ consents to participate was previously published [31, 32].

### nCounter® ion channel assay, (Nanostring technologies®, Seattle, USA)

Nanostring (nCounter assay) is an ultrasensitive technology that tests the gene expression via the molecular barcodes of the genes of interest that are directly counted with high accuracy. The reaction includes a reporter tag, capture tag, target-specific probes (transportome genes), and target molecules that hybridize to one another. The reporter tag carries a signal and the capture tag contains biotin that interacts accordingly with streptavidin. This reaction does not entail any amplification; it directly counts the already present mRNA copies. Here, the PDAC-relevant transportome genes (*n* = 101) were investigated in both HS and ML phenotypes of A818–6 cell line using nCounter assay (Supplementary Table 1). Prof Ivana Novak kindly helped choosing the PDAC-relevant transportome genes from the cell microarray’s significantly regulated genes and literature. Messenger RNA from the respective cell lines was isolated via Qiagen RNeasy kit. RNA was set to the concentration of 100 ng of purified total RNA in 30 μL reaction volume. nCounter analysis used 8 negative controls were the mean –in addition to the value of (2) as a standard deviation – were subtracted from samples. The samples were also normalized to the geometric mean of 6 positive controls in addition to 6 different housekeeping genes. The nSolver™ software (Nanostring Technologies) was used for analysis. Since precision of the analysis increases with expression level (counts), all the genes with an mRNA level below 30 counts were excluded from further analysis and a fold change cut-off of ≥1.5 or ≤ − 1.5 was also implemented.

### Statistics

For the cell microarray, HS versus ML comparison results were filtered first by fold change then by *p*-value (done by T-Test with unequal variance, unpaired) and corrected via Benjamini-Hochberg method. The significant differential expression values between the HS and ML together with the gene annotation were loaded in Microsoft Access Engine 2010 in order to create a database. The gene expression profiles of both phenotypes were compared to one another, where the HS expression was correlated to ML as control. After normalization, a number of 10,080 altered genes were detected in HS in correlation to ML. Later, a fold change of ≥4 or ≤ − 4 was applied and a *p*-value cut-off of ≤0.05 and finally Benjamini-Hochberg correction of ≤0.05 was applied. The whole-genome gene expression database was then screened for a gene list comprising 838 Transportome genes (Supplementary Table 2). This list was defined according to IUPHAR-DB [33], “Guide to Receptor and Channels” [34], and HUGO Gene Nomenclature Committee [35]. The description of the transportome gene list was previously described [32]. The differential expression values extracted for the 838 transportome genes were further validated by setting an adjusted *p*-value of ≤0.05 (Benjamini, and Hochberg correction) and a fold change (FC) ≤ − 2 or ≥ 2 for the both the cell and tissue microarrays. For the tissue microarray the statistics was computed using Limma R/Biocondoctor package [36] by applying a linear model as a statistical methodology (Supplementary Table 3) [37].

### Bioinformatics analysis

The differentially regulated genes were profiled using several online freely- available bioinformatics tools. Primarily, the gene lists were compared using WebGestalt (http://bioinfo.vanderbilt.edu/webgestalt) [38, 39] and a multiple gene list feature enrichment analyser ToppCluster (https://toppcluster.cchmc.org/) [40]. To investigate EMT features we used dbEMT (http://dbemt.bioinfo-minzhao.org/) gene resource [41]. Moreover, Venny 2.1 software was used to find the overlapping genes between the gene lists [42]. Finally, some of the predicted analyses were validated via pancreas expression database, PED [43–46] where the inquired gene lists were compared to a database of previously conducted experiments between PDAC patients versus healthy donors. Furthermore, SPEED [(S)ignaling (P)athway (E)nrichment using (E)xperimental (D)atasets] enrichment algorithm (http://speed.sys-bio.net) was used to specifically investigate the involvement of JAK-STAT, MAPK-PI3K, MAPK-only, TGFβ and TNFα in both A818–6 forms [47]. Also, KEGG mapper (https://www.genome.jp/kegg/mapper.html) was used to identify the involved pathways. Gene set enrichment analysis (GSEA) was used to interpret the microarray data by assigning each gene to its specific biological function and distinct pathways [48]. The analysis is based on Gene Ontology (GO) annotation system [49]. This method implements the hypergeometric distribution to calculate the probabilities that a biological attribute is overrepresented in a gene data set.

## Results

### HS/ML as a differentiation model

**Fig. 1:**
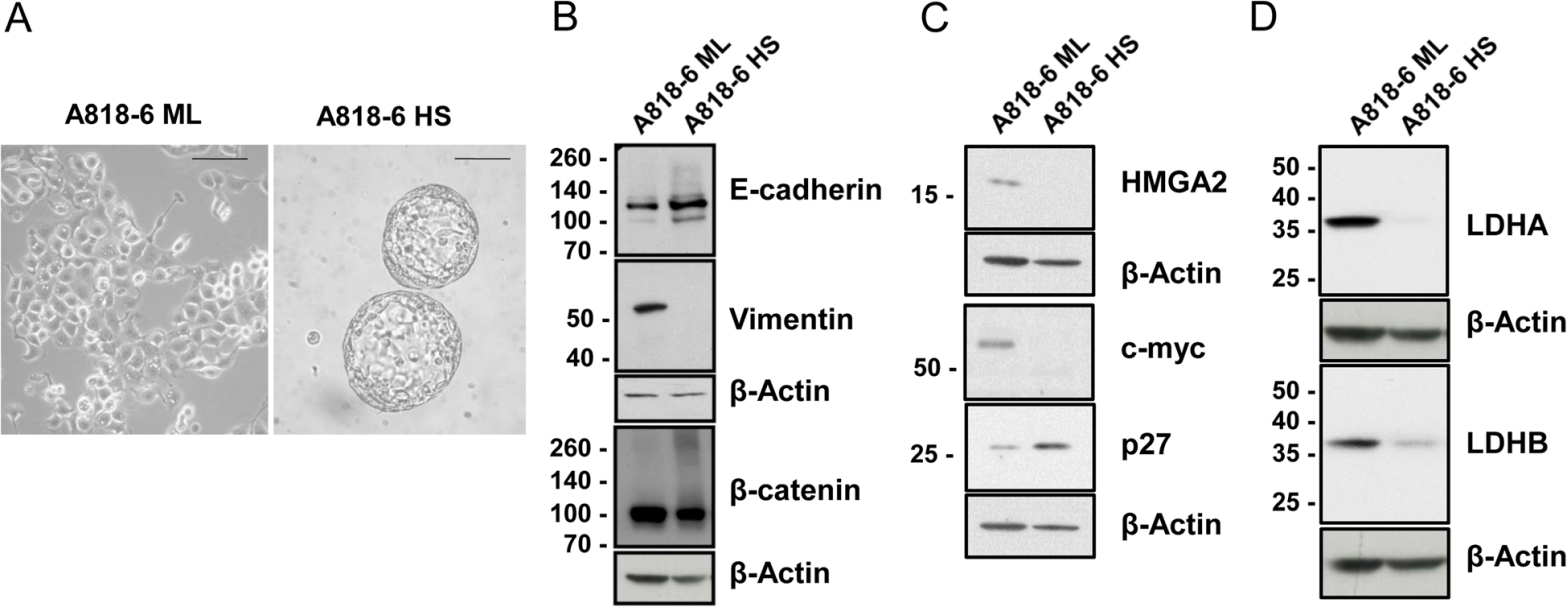
Characterisation of ML and HS cells with regards to morphology, EMT and metabolic markers. **a** bright field light microscopy of both A818–6 forms 2D monolayer (ML) and 3D hollow spheres (HS) [scale line = 100 μM]. The protein levels of some EMT markers [E-cadherin, vimentin, β-catenin] **b** also the protein levels of HMGA2, c-myc and p27 as proliferation markers **c** and the levels of some metabolic markers **d** were detected in the whole cell lysate via immunoblotting. Beta actin was used as a loading control

Though cellular plasticity enables normal cells to maintain homeostasis, it is also responsible for the capability of epithelial tumour cells to invade and metastasize [50]. It has been proposed that cancer cells, via activation of the epithelial-to-mesenchymal transition (EMT), gain the ability to migrate and invade distant organs. Contrariwise, mesenchymal-to-epithelial transition (MET) must occur in these cells for successful colonization of the new tissue [51]. Fittingly, the HS/ML in vitro system of the A818–6 PDAC cell line represents a unique system to study these transitions. The HS/ML system was presented as a model for studying the differences between a malignant/undifferentiated (ML) and a quasi-normal/differentiated pancreatic (HS) epithelium [27–29] (Fig. 1a). To validate the differentiation/dedifferentiation status, the protein levels of two markers were compared in both forms. Consistent with the more differentiated, epithelial character of cells growing as HS, Western blot analyses showed clearly higher level of E-cadherin than in ML cells, whereas the expression of the mesenchymal marker vimentin was restricted to ML cells (Fig. 1b).

In agreement with the previously described lower proliferative activity of HS, the negative cell cycle regulator p27 was strongly increased in HS. Also, consistent with the malignant phenotype, proteins enhancing cell proliferation like HMGA2 and c-myc were strongly decreased in HS in comparison to ML cells (Fig. 1c). In addition, protein levels of lactate dehydrogenase protein isoforms A and B (LDHA, LDHB), key enzymes participating in glucose metabolism and overexpressed in pancreatic cancer were highly expressed in ML and severely down regulated in HS (Fig. 1d).

**Fig. 2:**
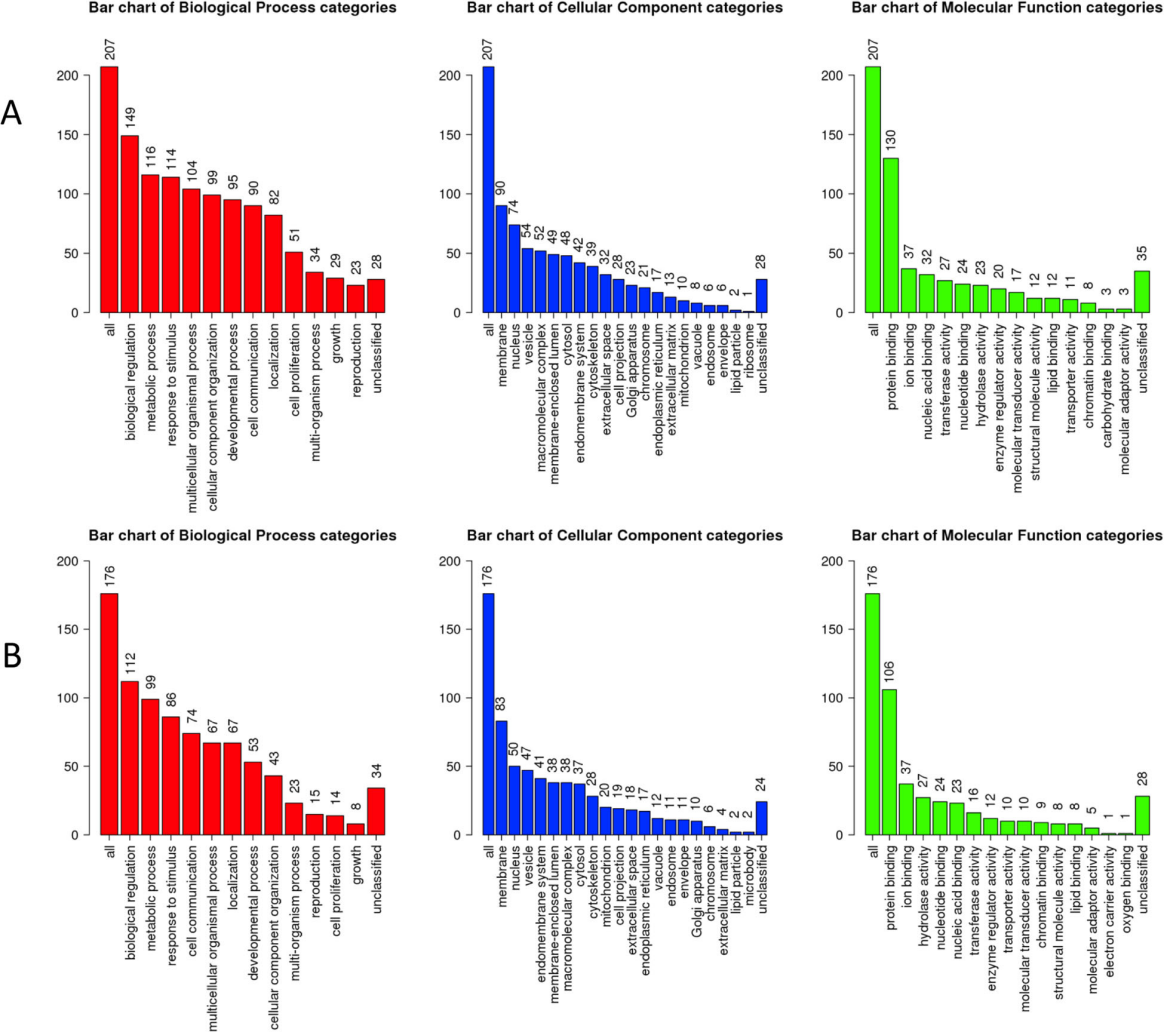
WebGestalt analysis of the different Gene Ontology terms in HS/ML from the cell microarray data. Bar chart showing the number of genes from the cell microarray that are involved in the different Gene Ontology terms as predicted by the Gene Set Enrichment Analysis (GSEA) via WebGestalt. **a** Gene Ontology terms of the ML upregulated genes, **b** Gene Ontology terms of the HS upregulated genes. The graph is showing the number of genes involved in the different biological processes (Red), Cellular components (Blue) and Molecular functions (Green)

**Fig. 3:**
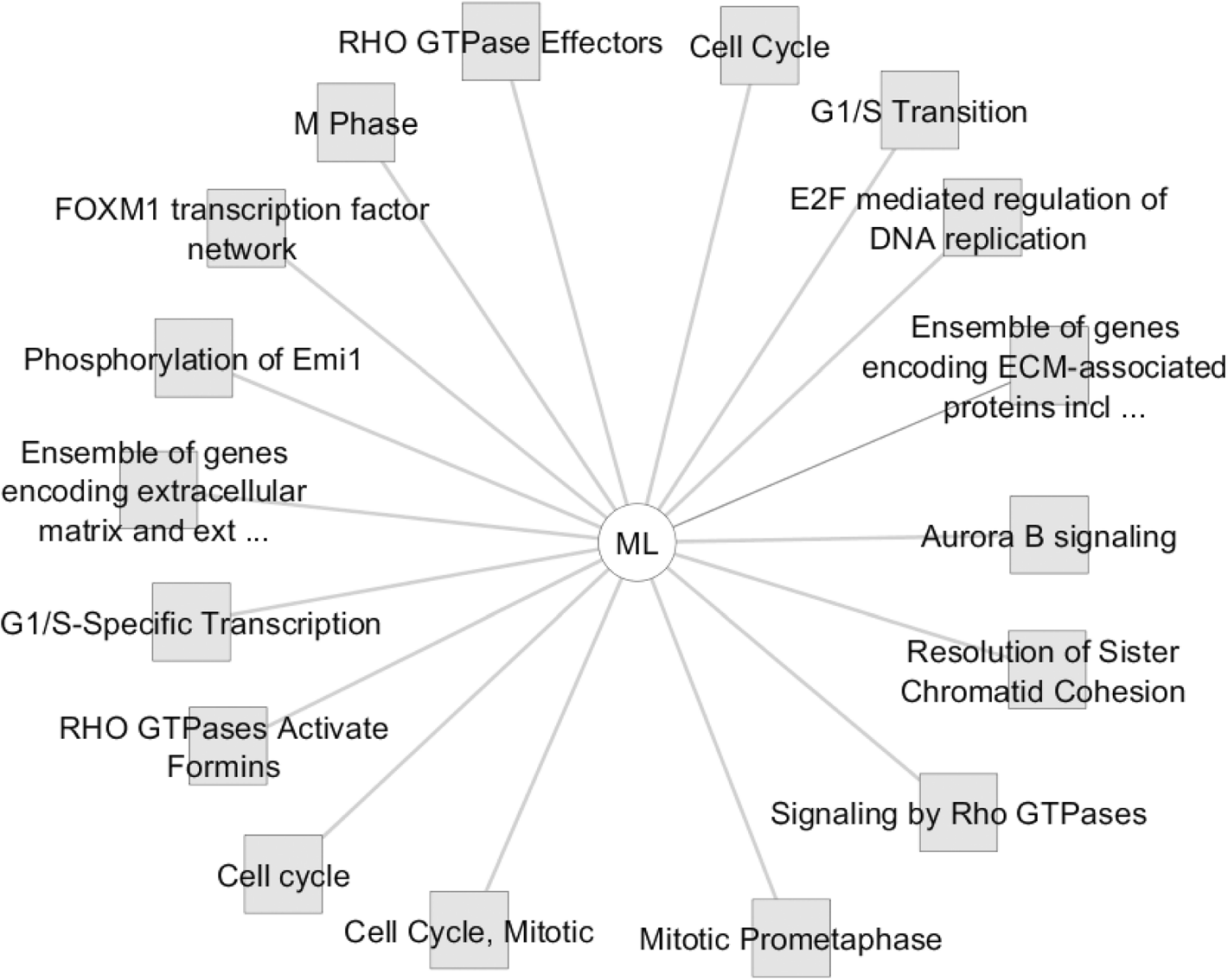
Toppcluster analysis of the activated pathways in HS/ML from the cell microarray data. The pathways possibly regulated by the two sets of differentially regulated genes in the cell microarray of HS/ML system as predicted by Topplcuster. Cytoscape software was used as a visualization tool to build the gene expression network

**Fig. 4:**
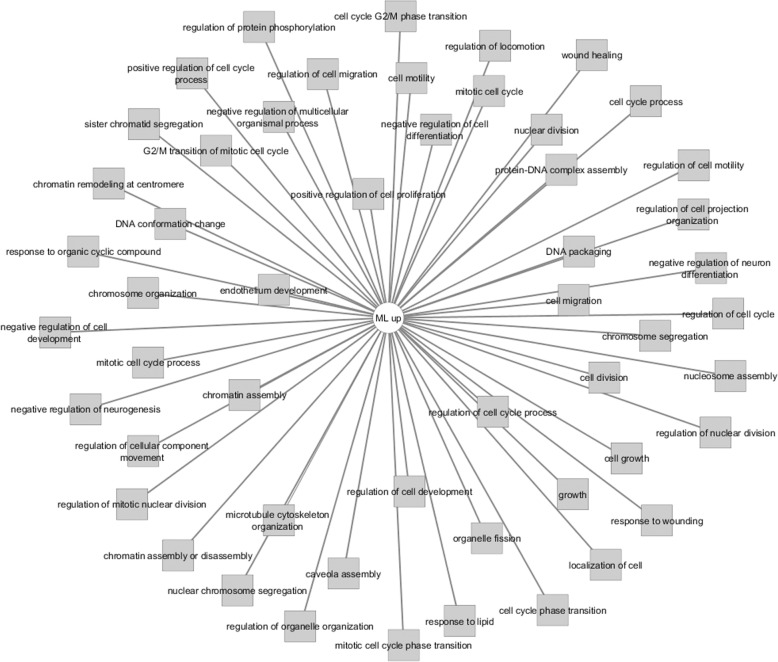
Toppcluster analysis of biological processes in HS/ML from the cell microarray data. Biological processes possibly involved in the regulation of the differentially regulated genes in the cell microarray of the HS/ML system as predicted by Topplcuster. However, many were affected by the ML dysregulated genes. Cytoscape software was used as a visualization tool to build the gene expression network

**Fig. 5:**
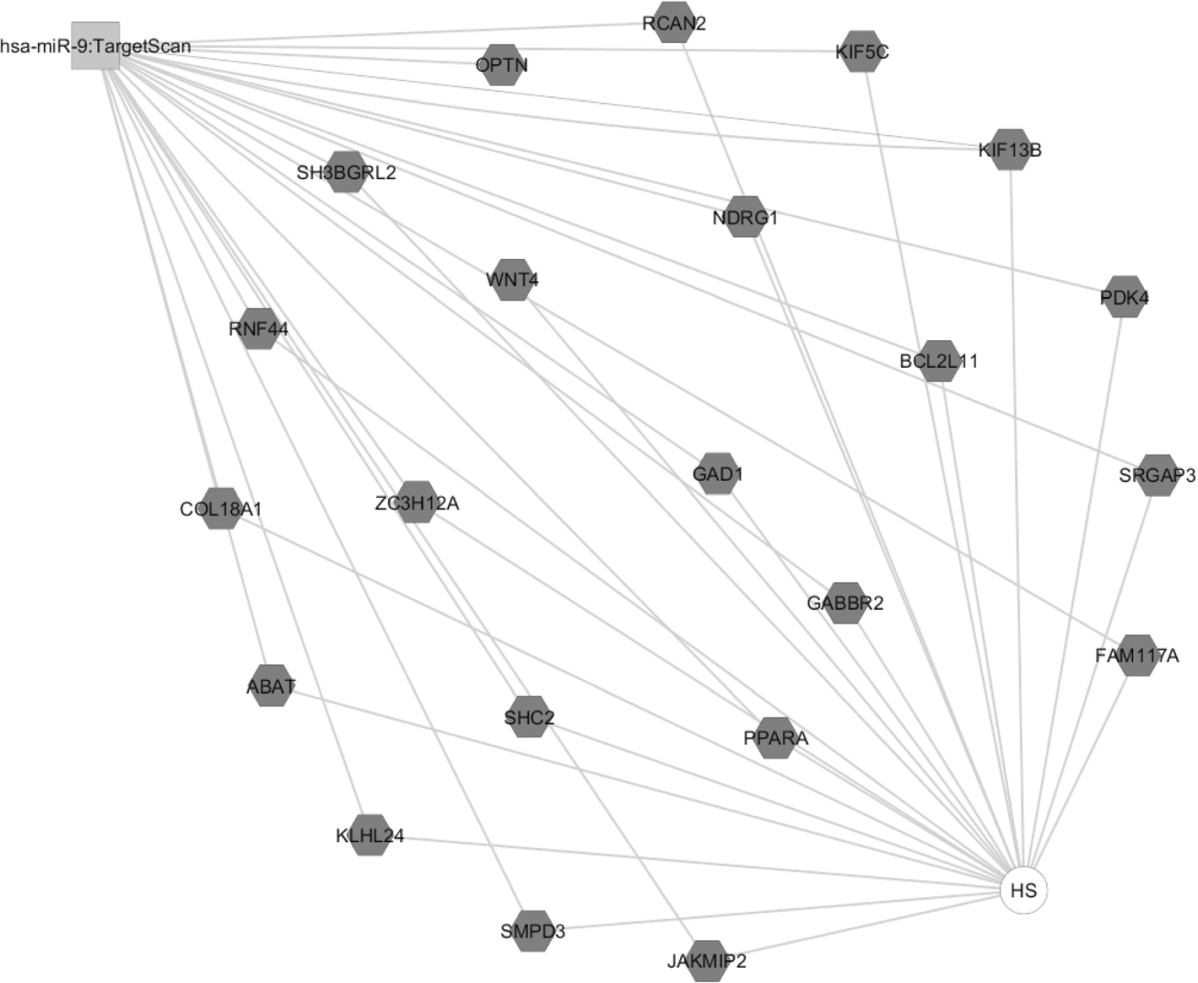
Toppcluster predictions of the possible miRNA involvement in HS/ML from the cell microarray data. The miRNA possibly involved in the regulation of the genes modulated in the cell microarray in both the ML and HS as predicted by Topplcuster. Interestingly, Toppcluster could only predicted one miRNA (hsa-miR-9) that could modulate multiple genes that are overexpressed in HS and non for ML. Cytoscape software was used as a visualization tool to build the gene expression network

**Fig. 6:**
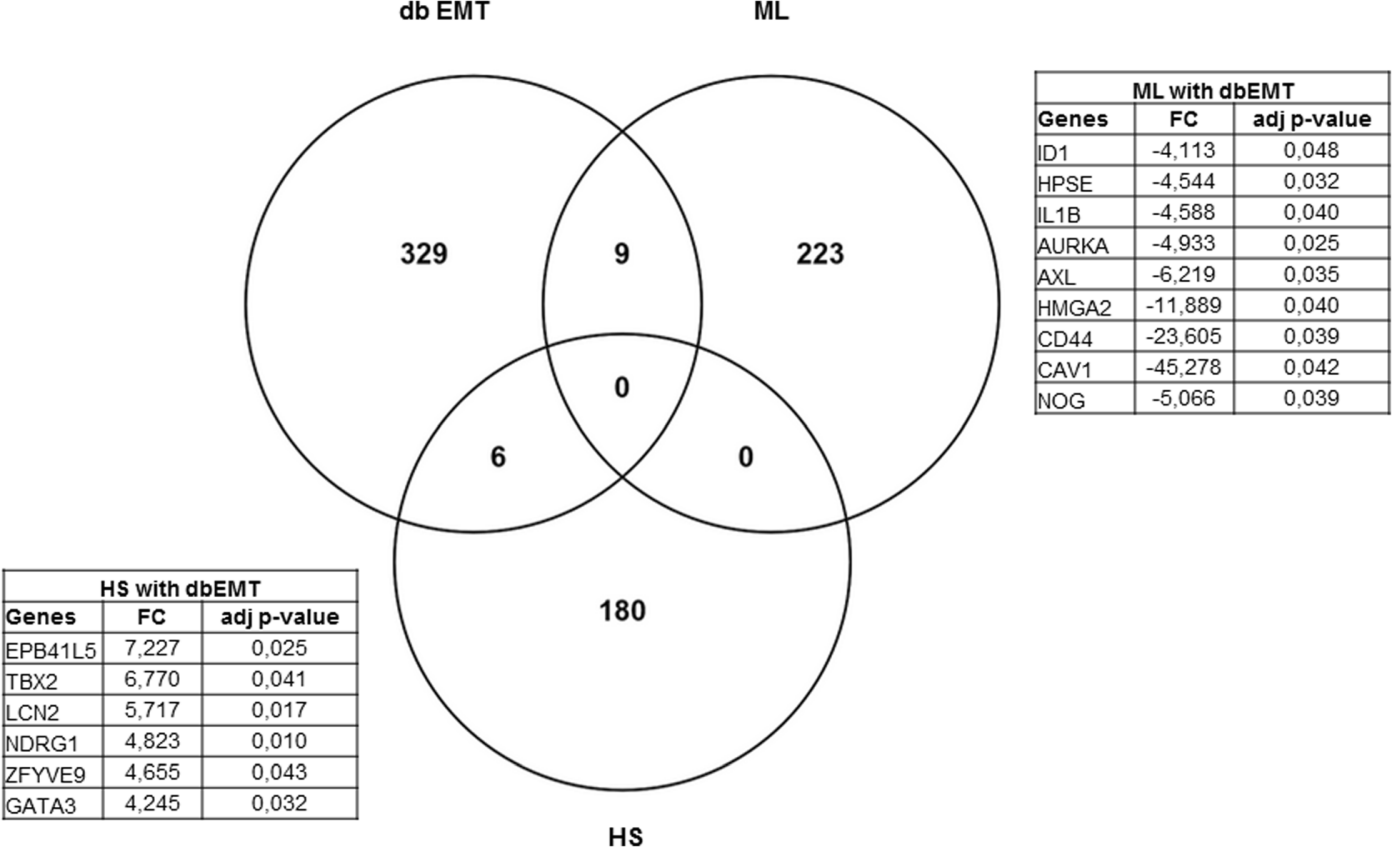
Prediction of the involvement of EMT in HS/ML from the cell microarray data. Venn diagram showing the number of genes involved in EMT in both ML and HS from the cell microarray. Table (upper right) showing the fold change (FC) and *p*-values of the upregulated EMT genes in ML and (Lower left) table is showing those genes in HS

In an attempt to explore the possible molecular drivers for (de)differentiation in these cells, we have performed an automated genome wide gene expression microarray (cell microarray) analysis using mRNA extracts from both A818–6 cell phenotypes. This analysis resulted in a total of 424 significantly regulated genes of which 187 and 237 genes were upregulated in HS and ML, respectively (Supplementary Table 4). Initially, the evaluation was enriched by correlating the differentially regulated genes with Pancreatic Expression Database [PED [43–46];] as a platform for gene expression studies of the pancreatic cancer (Supplementary Tables 5, 6). PED analyses confirmed the association of a higher number of genes upregulated in ML (*n* = 26) with PDAC development in comparison to healthy epithelium, thus denoting ML’s more malignant nature in comparison to its HS (*n* = 7) counterpart. Moreover, consistent with lower proliferation rate of cells growing as a HS, both PED and WebGestalt revealed the association of multiple genes overexpressed in ML in cell cycle and proliferation in PDAC (Fig. 2a), conversely, much less number of genes overexpressed in HS were involved in these processes (Fig. 2b & Supplementary Tables 5, 6, 7 and 8). Additionally, Toppcluster revealed a correlation between the genes which were upregulated in ML not only with cell cycle, growth and division but also with cell motility (Figs. 3 and 4). Similar results were obtained from Reactome (via WebGestalt, Supplementary Tables 7, 8). Obviously there were no predicted pathways or biological processes for HS altered genes in Toppcluster. However, many pathways were affected by the ML dysregulated genes. Interestingly, Toppcluster showed that 22 genes of the upregulated group in HS were all targets of microRNA *miR-9* (Fig. 5). Additionally, and in agreement with the more differentiated, more benign phenotype of HS, the cell microarray displayed a number of 9 EMT-related genes which were upregulated in ML as compared to 6 in HS. These results were obtained by blotting the cell microarray results against the genes known to be involved in EMT as published in dbEMT database (Fig. 6). Altogether, and in line with our previous data [27–29], these molecular differences between HS and ML confirmed that A818–6 cells are able to switch between differentiated/quasi benign state and undifferentiated/malignant state thus providing a good system that allows the analysis of the genetic mechanisms driving and sustaining this process.

### HS/ML system as a model to study the transportome in PDAC

As formerly described, the epithelial cellular polarity plays an important role in maintaining cellular volume and acid/base transport in pancreatic ductal cells [15], and the involvement of the transportome in PDAC development was also previously reported [17, 23, 52–54]. Noteworthy, the 3D orientation of HS was previously shown to allow the A818–6 cells to regain relatively normal epithelial polarity which was in turn lost when the HS were disrupted and ML reformed and vice versa [27, 29]. Earlier, mucin− 1, a marker of the apical border of the normal pancreatic epithelial cells, and β-catenin a basolateral marker were utilized to stain HS and ML cells. It was shown that HS regained an inverted polarity where HS displayed an outer apical border and an inner basolateral border stained with mucin− 1 and β-catenin, respectively. HS showed also positive staining of the differentiation marker carbonic anhydrase II (CA II) and secreted carcinoembryonic antigen-related cell adhesion molecules (CEACAMs) into the supernatant around them, both of which denote the polarization of HS in comparison to ML [27, 29]. Collectively, the HS/ML in vitro system could provide a distinctive insight into the involvement of the transportome during the differentiation/dedifferentiation process of A818–6 cells.

In order to study whether the changes in differentiation status between HS and ML correlate with changes in the expression level of transportome genes, two different approaches were implemented. Firstly, the above-mentioned cell microarray dataset was screened for 840 known transportome genes and additionally, a Nanostring nCounter analysis of HS/ML cells was performed. In the cell microarray, among the differentially regulated genes, we found 126 transportome genes; 68 of which were overexpressed and 58 down-regulated in HS compared to ML (Supplementary Table 9). In addition, a custom-made Nanostring nCounter array screening for the important and significantly altered PDAC-relevant expression of transportome genes was performed. The nCounter array included a number of 101 PDAC-related transportome genes, which were identified from both the cell microarray and literature. The final nCounter analysis of the transportome gene expression included 25 up- and 22 down-regulated genes in HS compared to ML (Table 1). From both, the cell microarray and the nCounter assay a number of consistently upregulated (*n* = 11) as well down-regulated (*n* = 15) transportome genes were found in HS when compared with its corresponding ML (Table 2). Thus, both approaches underline the involvement of transportome in this (de)differentiation model.

**Table 1:** Altered genes between ML and HS according to nCounter assay (mean of two experiments)

Probe Name	HS vs ML
KCNF1	67,465
SCNN1B	29,385
KCNQ1	9,87
SLC7A2	6,68
CACNA1D	5,94
TRPV6	3535
TNFSF10	3385
SLC4A4	3035
SLC26A11	2745
SLC1A4	2,53
ATP6V0A1	2085
SLC41A1	2,03
ATP6V0E2	1,95
RPL13A	1935
P2RX4	1,85
TNFRSF10B	1,84
KCNK1	1,76
KCNK6	1,72
SLC26A6	1705
ATP6V1C1	1,7
HCN3	1655
KCNK15	1,64
P2RX5	1,63
LRRC8A	1,52
ATP6V0E1	1515
ANO1	− 1575
PBGD	− 1635
TNFRSF10A	−1,76
SLC9A1	-1,82
ABCG1	-1,85
SLC1A1	− 2025
SLC38A6	−2,19
GJC1	− 2255
GJB2	−2,63
SLC16A7	−2,7
ADORA2B	− 2705
SLC20A1	−2,93
CA12	−3,09
LDHB	−3,12
NT5E	−3,6
LDHA	− 4095
GJB5	−5,07
SLC29A1	− 5135
GJB4	− 5605
GJB3	−5,9
SLC16A1	−20,05
GJA5	−20,69

**Table 2:** Common altered transportome genes in HS versus ML in both nCounter and cell microarray

Gene Symbol	Description	FC nCounter	Cell microarray	
		mean of 2 exp.	FC	adj. *p*-value
KCNF1	potasium voltage-gated channel, subfamily F, member 1	67.465	475.04	0.03
SCNN1B	sodium channel, no volateg-gated 1, beta	29.385	47.45	0.034
KCNQ1	potasium voltage-gated channel, KQT-like subfamily, member 1	9.87	8.59	0.012
SLC7A2	solute carrier family 7 (cationic amino acid transporter, y + system)	6.68	10.83	0.028
CACNA1D	calcium channel, voltage-dependant, L-type, alpha 1D subunit	5.94	5.05	0,008
TRPV6	transient receptor potential cation channel, subfamily V, member 6	3.535	5.37	0.035
SLC4A4	solute carrier family 4, sodium bicarbonate cotransporter, member 4	3.035	34.41	0.017
SLC26A11	solute carrier family 26, member 11	2.745	2.48	0.003
SLC1A4	solute carrier family 1 (glutmate/neutral amino acid transporter, member 4	2.53	4.16	0.026
SLC41A1	solute carrier family 41, member 1	2.03	2.24	0.008
HCN3	hyperpolarization activated cyclic nucleotide-gated potassium channel 3	1.655	2.09	0.005
ABCG1	ATP binding cassette, subfamily G (WHITE), member 1	−1.85	− 5.51	0.008
SLC1A1	solute carrier family 1 (neuronal/epithelial high affinity glutmate transporter, system Xag), member 1	−2.025	−2.51	0.027
SLC38A6	solute carrier family 38, member 6 (SLC38A6), transript variant 2	−2.19	−3.5	0.018
GJC1	gap junction protein, gamma 1, 45 kDa	−2.255	−2.83	0.007
GJB2	gap junction protein, beta 2, 26 kDa	−2.63	−2.34	0.006
SLC16A7	solute carrier family 16, member 7 (monocarboxylic acid transporter 2; MCT2)	−2.7	−4.16	0.0034
SLC20A1	solute carrier family 20, (phosphate transporter), member 1	−2.93	−2.03	0.045
LDHB	lactate dehydrogenase B	−3.12	−2.59	0.008
LDHA	lactate dehydrogenase A, (LDHA), transcript 1	−4.095	−3.83	0.005
GJB5	gap junction protein, beta 5, 31.1 kDa	−5.07	−5.2	0.008
SLC29A1	solute carrier family 29 (nucleoside tranaporter), member 1, mitochondrial protein	−5.135	− 5.46	0.008
GJB4	gap junction protein, beta 4, 30.3 kDa	−5.605	−3.78	0.012
GJB3	gap junction protein, beta 3, 31 kDa	−5.9	−5.3	0.015
SLC16A1	solute carrier family 16, member 1 (monocarboxylic acid transporter 1; MCT1)	−20.05	−52.8	0.021
GJA5	gap junction protein, alpha 5, 40 kDa	−20.69	−16.26	0.008

**Fig. 7:**
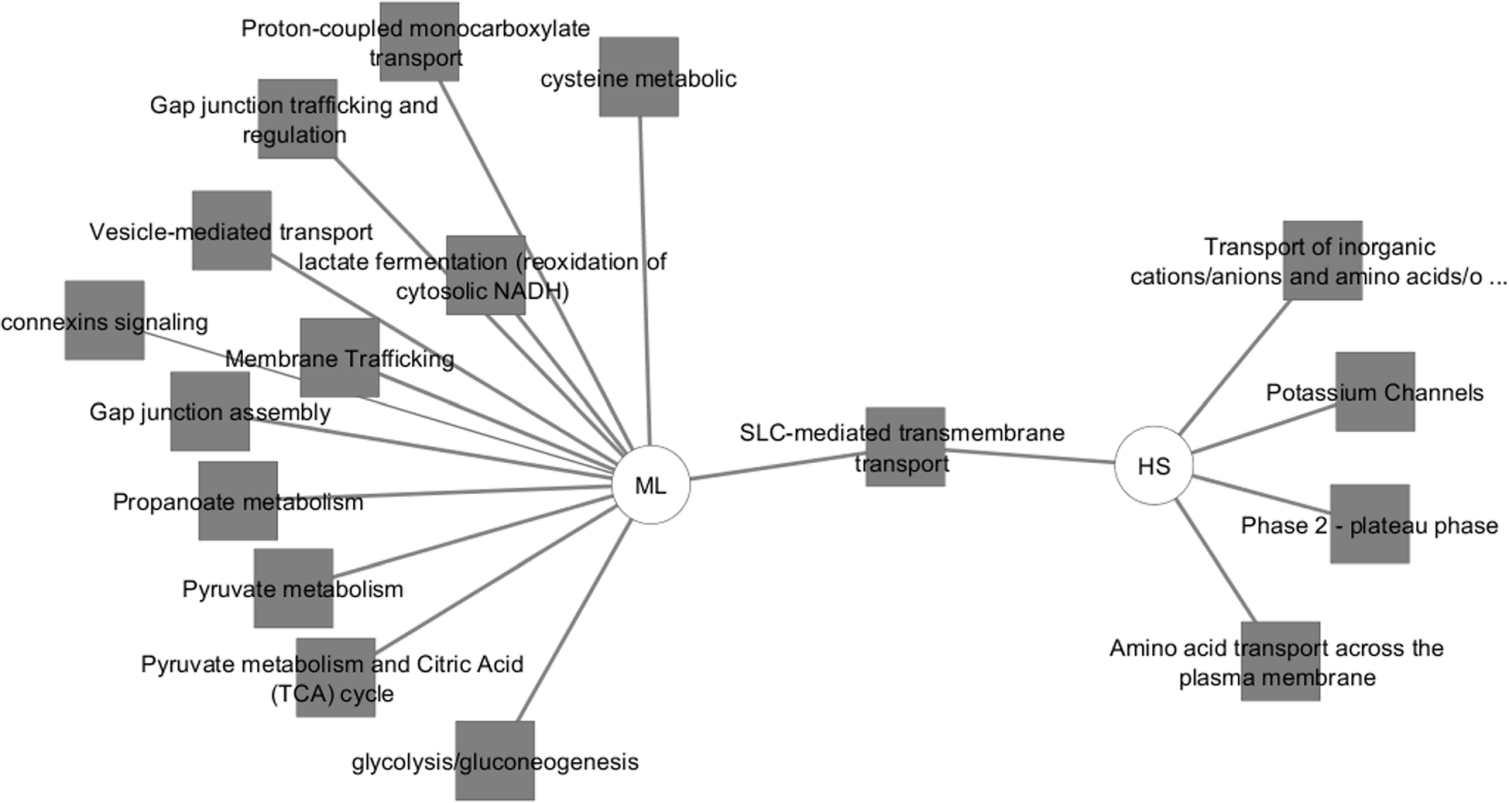
Toppcluster analysis of the activated pathways in HS/ML in response to the altered transportome. The pathways possibly altered by the differentially regulated Transportome genes in HS/ML system from both the cell microarray and nCounter analyses as predicted by Topplcuster. Cytoscape software was used as a visualization tool to build the gene expression network

**Fig. 8:**
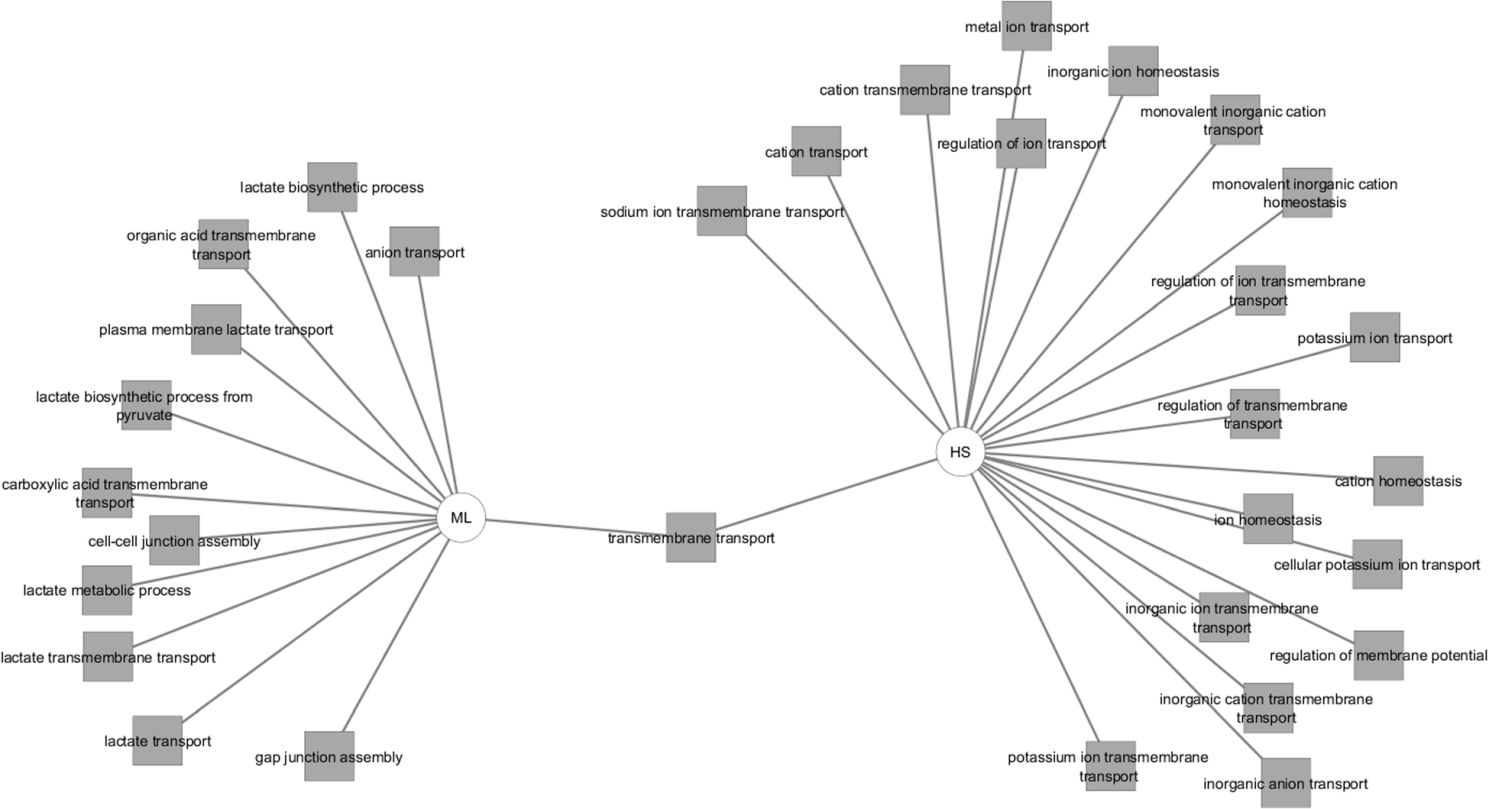
Toppcluster analysis of the biological processes in HS/ML in response to the altered transportome. The biological processes possibly altered by the differentially regulated Transportome genes in HS/ML system from both the cell microarray and nCounter analyses as predicted by Topplcuster. Cytoscape software was used as a visualization tool to build the gene expression network

**Fig. 9:**
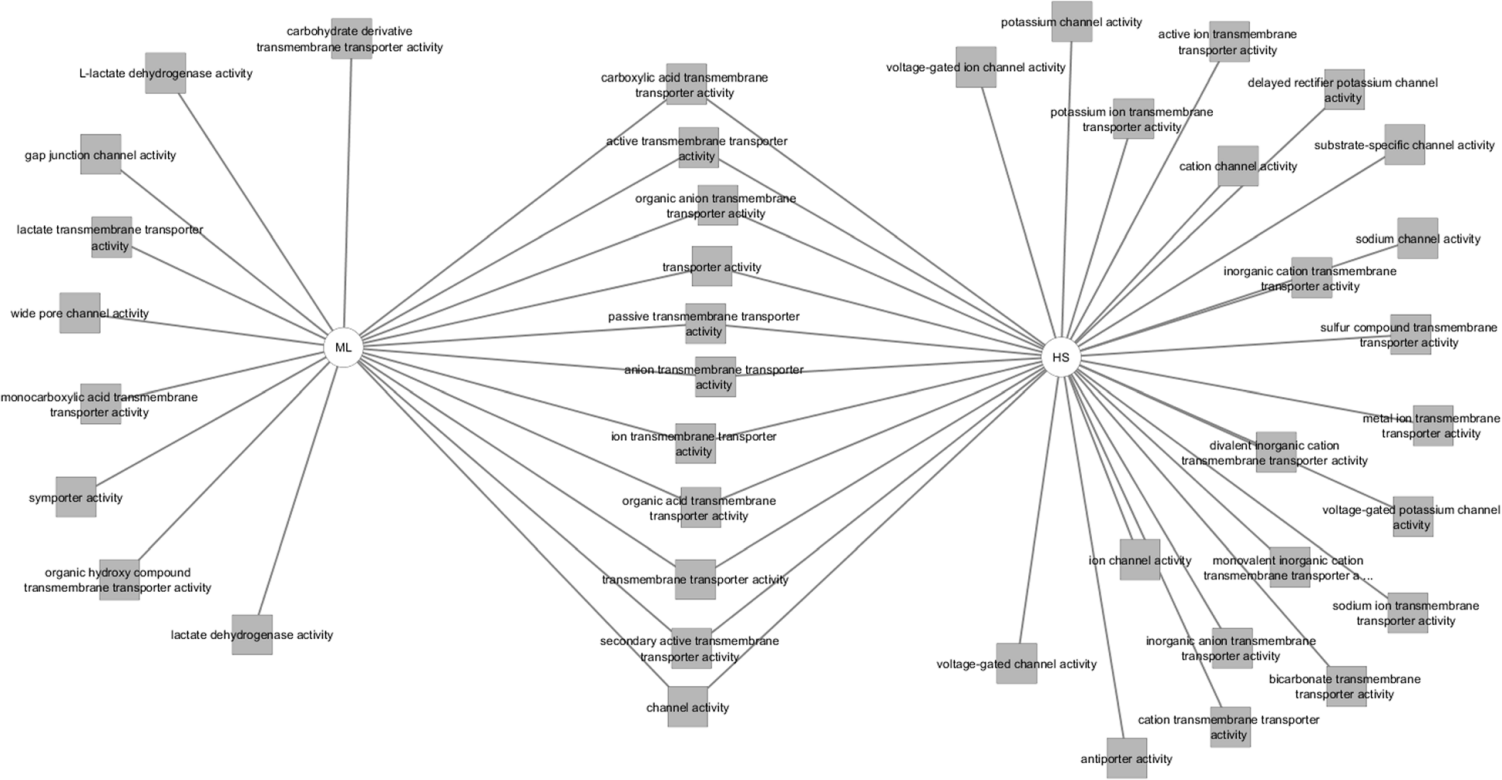
Toppcluster analysis of the molecular functions in HS/ML in response to the altered transportome. The molecular functions possibly altered by the differentially regulated Transportome genes in HS/ML system from both the cell microarray and nCounter analyses as predicted by Topplcuster. Cytoscape software was used as a visualization tool to build the gene expression network

This transportome gene list was uploaded to Toppcluster and a comparison was created between the up and down-regulated genes in terms of pathways, biological processes, molecular functions and the possible miRNA regulators of those genes (Figs. 7, 8 and 9). Regarding the predicted pathways, only one pathway was commonly altered with both the up- and down-regulated genes in HS cells (SLC-mediated transmembrane transport). Specifically, potassium channel genes, genes related to amino acid, oligopeptide transport as well as transport of inorganic cations/anions were enriched in HS in comparison to ML. Conversely, gap junction assembly, metabolism, membrane trafficking and vesicle-mediated signalling pathways were upregulated in ML in comparison to HS. Among the biological processes influenced by the upregulated genes in HS were several ion transport proteins (sodium, potassium, metal) whereas, the down-regulated genes were associated with cell-cell junction assembly, lactate biosynthetic processes and anion transport. Furthermore, the overexpressed transportome genes in HS were involved in molecular functions including; voltage-gated ion channel activity, substrate specific channel activity and antiporter activities among others. On the contrary, the down-regulated transportome genes are those involved in the control of other molecular functions as wide-pore channel activity, symporter activity and lactate dehydrogenase activity. Finally, both the nCounter and cell microarray analyses confirmed the higher expression levels of both metabolic enzymes, LDHA and LDHB, in ML cells compared to HS. Worth mentioning, though Toppcluster is a valuable tool that allows the comparison between different gene groups, it only lists all the possible functions/pathways shared by the investigated genes and it does not specify which is more relevant to the current gene clusters.

To further authenticate our hypothesis that the HS/ML system is a valid system to study the role of transportome in the de-differentiation process in PDAC, we compared the above described results with our recently reported transportome analysis in PDAC patients’ tissues [31, 32]. Previously, we compared the gene expression profile of 19 PDAC patients’ malignant pancreatic microdissected tissue specimens (tumour epithelium) with 13 different normal pancreatic epithelial specimens (normal epithelium) that were performed using a 133 A/B Affymetrix microarray technology (tissue microarray). Among the 616 significantly altered genes, 63 were transportome genes of which genes were overexpressed and 45 suppressed in PDAC. The dysregulated genes were not only involved in pH regulation, the control of the cellular volume, secretion and polarity but also in cellular differentiation [32]. In the current study, we compared these tissue microarray results with our data from both the cell microarray and the nCounter array. This comparison confirmed the differential expression of numerous transportome genes in PDAC and ML in relation to normal epithelium and HS, respectively (Table 3). This comparison identified a number of 3 upregulated genes (GJB2, GJB5 and SLC38A6) in PDAC and ML, and another 3 transportome genes (KCNQ1, SLC4A4 and TRPV6) which were correlated with normal pancreatic epithelium and HS. To predict the role of these differentially regulated transportome genes, a bioinformatics analysis was performed using publicly available open source databases. This part of the analysis showed the enrichment of the pancreatic secretion (KCNQ1, SLC4A4), the epithelial fluid secretion (TRPV6) and the maintenance of bicarbonate transport (SLC4A4) in the normal pancreatic epithelium and HS. Additionally, it was found that some PDAC and ML-related transportome genes were involved in metabolic transport (SLC38A6) and intercellular communication and cell adhesion (GBJ2, GJB5). Correspondingly, the transportome genes in normal pancreatic epithelium and HS were functionally related to transepithelial ion and fluid secretion, where bicarbonate and/or chloride transport were enriched via SLC4A4, transepithelial transport and setting membrane potential via KCNQ1, cAMP and intracellular calcium signalling via TRPV6. Although the current study analysed the transportome gene expression profiling, it did not investigate the overall functions of ion channels. All in all, this study introduces the HS/ML model as a valid system to study the differential regulation of transportome genes in both differentiation and PDAC development as well as during this transition.

**Table 3:** Common altered transportome genes in HS/ML and Tumor epithelium (TE)/normal epithelium (NE) in both nCounter and array data

Gene Symbol	Description	FC nCounter	HS/ML (cell microarray)		TE/NE (tissue microarray)	
		mean of 2 exp.	FC	adj. p-value	FC	adj. *p*-value
KCNQ1	potassium voltage-gated channel, KQT-like subfamily, member 1	9.87	8.59	0.012	−2.11	0.003
TRPV6	transient receptor potential cation channel, subfamily V, member 6	3.535	5.37	0.035	−5.36	0.001
SLC4A4	solute carrier family 4, sodium bicarbonate cotransporter, member 4	3.035	34.41	0.017	−4.92	0.001
SLC38A6	solute carrier family 38, member 6 (SLC38A6), transript variant 2	−2.19	−3.5	0.018	2.33	0.004
GJB2	gap junction protein, beta 2, 26 kDa	−2.63	−2.34	0.006	3.90	0.007
GJB5	gap junction protein, beta 5, 31.1 kDa	−5.07	−5.2	0.008	3.60	0.013

## Discussion

In the current study, we aimed to investigate the differences between the two A818–6 cell forms, ML and HS and to test whether the HS/ML (de)differentiation model could be useful in studying the role of transportome in PDAC development. Therefore, a whole-genome cell microarray in addition to the nCounter analyses was performed to investigate the transportome genes that may play a role in the process of malignant transformation. The expression data showed that the two forms of A818–6 are strictly distinctive. On the one hand, the 2D ML cells are more aggressive and exhibiting mesenchymal features. On the other hand, the HS structures show less aggressiveness with more epithelial characteristics. This degree of plasticity provided these cells with cancer stem cell properties that drove them in a series of transformations between the epithelial and mesenchymal cell types [51]. These properties were confirmed via detection of protein levels of respective markers and bioinformatic analyses of the data from the two arrays. Known epithelial marker like E-cadherin (CDH1) and alpha catenin (CTNNA1) and keratin 15 (KRT15) were restored in HS. However, markers like HMGA2, CD44, Caveolin 1 and the mesenchymal marker vimentin were boosted in ML. HMGA2 is a known transcriptional regulator that facilitates the transcription of many other pro-tumoural genes and it was previously linked with shortened survival in PDAC patients [55]. CD44 plays not only a role in EMT but also is a dedifferentiation marker that has been formerly reported to be highly expressed in anaplastic lesions and is correlated with cancer stem cells in PDAC [56, 57] and Caveolin 1 which was formerly suggested to be considered as an aggressiveness marker in PDAC [58]. Intriguingly, T-box transcription factor 2 (TBX2) gene, which is a key player in the development of the embryo and its overexpression has been associated with several malignancies including PDAC, was also upregulated in HS [59]. In other words, the mesenchymal characteristics of ML and the possible involvement of EMT is not entirely correlated with metastasis, as was previously confirmed in lung cancer and PDAC [60, 61]. Otherwise these two cell forms could represent two intermediate cell types on the scale of EMT transition.

Interestingly, we found that 22 upregulated genes in HS are targets of *miR-9*. This may denote that either the level of this microRNA is boosted in cells grown as ML or that miR-9 is actively deregulated in HS and thus implicating *miR-9* – oncomir – in PDAC malignant development. The role of *miR-9* in malignant transformation is not clear so far. On the one hand, *miR-9* level is upregulated and reported to be involved in malignant progression of both hepatocellular [62] and prostatic carcinoma [63]. On the other hand, *miR-9* level is regarded as a tumour suppressor microRNA in breast cancer, where its induction leads to anti-proliferative, anti-invasive and pro-apoptotic effects [64]. However, it was also implicated in the promotion of neovascularization [65]. Here, our system suggests a role of microRNA *miR-9* in inducing the mesenchymal features in A818–6 ML cells.

Furthermore, we investigated the involvement of the transportome genes in the malignant transformation in our HS/ML model. The overall analysis of the three arrays denoted the implication of some key transportome genes in PDAC malignant transformation. Among those genes that were overexpressed in PDAC tissues/ML was GJB2 that was previously found to be barely expressed in normal pancreatic ductal epithelium, while highly expressed [66] and correlating with poor prognosis in PDAC patients [66, 67]. GJB2 was also suggested as a prognostic marker in pancreatic cancer [68]. Moreover, the current analyses showed that the expression of TRPV6, SLC4A4 and KCNQ1 were down-regulated in PDAC/ML. This transcriptomic profiling of these transportome genes points out to the possible loss of differentiated secretory epithelial cells functions. Therefore, it can be concluded that the control of the resting membrane potential via KCNQ1, the vectorial bicarbonate transport via SLC4A4, as well as the epithelial fluid secretion via KCNQ1 and TRPV6 were inhibited in PDAC (tumour epithelium and ML), while maintained in the normal epithelium (normal epithelium and HS). Another function of, the calcium channel, TRPV6 in the normal pancreatic epithelium was the activation of programmed cell death [69], and the inhibition of TRPV6 resulted in cell survival in gastric cancer cells [70]. Under normal conditions, this calcium - permeable channel leads to a cytosolic calcium increase that results in apoptosis thus restoring the capability to control the elimination of the cells from the circulation. In other words, the down-regulation of TRPV6 in PDAC could aid the cancer cells to evade apoptosis. However, it was also recently found that TRPV6 gene was upregulated in some PDAC cells and its inhibition was correlated with decreased invasiveness and metastasis [71, 72]. In other tumour entities, TRPV6 was also correlated with high proliferation rate of the prostate and breast cancer cells, as the calcium conductance activates the calcium/ calmodulin/calcineurin dependent transcription factor NFAT affecting the expression of the cell-cycle regulators [73].

Another two transportome members (KCNQ1 and SLC4A4) were previously predicted to enhance pancreatic secretion. Generally, potassium channels were previously implicated in the development of cancer [74]. In the current study, only one down-regulated potassium channel gene, KCNQ1 (K_V_7.1), was detected in PDAC. Since, K_V_7.1 channel was annotated as a protein engaged in the control of cell volume, stabilization of membrane potential and maintenance of electrogenic epithelial electrolyte transport. Additionally, K_V_7.1 channel has been detected and characterized in many absorbing and secretory epithelia including the pancreatic ducts [75, 76]. In these tissues, the K_V_ channels enhance the potassium conductance in response to cell swelling or purinergic stimulation in the epithelial transport [76, 77]. The resulting potassium efflux stabilizes the resting membrane potential and supports chloride exit, which finally coordinates the electroneutral potassium, chloride secretion at the basolateral membrane [76]. Therefore, KCNQ1 has been previously proposed as a tumour suppressor gene [78], Rapetti-Mauss et al. also demonstrated a positive correlation between high KCNQ1 expression and well-differentiated epithelial cell lines and consequently with patient survival in primary stage of colorectal carcinoma [79]. In the same study, the authors described a progressive loss of KCNQ1 with increasing mesenchymal phenotype in poorly differentiated cells, as a consequence of repression of the KCNQ1 promoter.

The lower mRNA level of the cotransporter SLC4A4 in PDAC suggests that the pancreatic bicarbonate secretion and intracellular pH were dysregulated during PDAC development. SLC4A4 is an important protein in healthy pancreatic ducts, as the acid-base homeostasis is a key mechanism to control bicarbonate-rich fluid secretion [11]. As a bicarbonate transporter, SLC4A4 drives the transport of sodium and bicarbonate ions across the basolateral membrane of the pancreatic duct. The widely accepted secretory model describes the role of SLC4A4 in the vectorial transport of bicarbonate from the interstitium to the duct [11, 80]. SLC4A4 expression was formerly found to be down regulated in PDAC [81]. It should be noted that the cystic fibrosis transmembrane conductance regulator (CFTR) gene, the principal channel controlling fluid transport in secretory cells [13, 74, 82], did not show any significant change between HS and ML cells. Consequently, the current study could show a clear difference in some key transportome genes’ expression that could be implicated in differentiation/dedifferentiation process in PDAC.

To date, there are several other 3D models that involve PDAC cell lines but each serves a distinct aim. The first 3D PDAC model was the A818–6 HS/ML system and it was introduced in 1999 [27–29] to investigate the process of differentiation in the pancreatic epithelium. Another 3D model was developed using normal pancreatic ductal epithelial cell with a KRAS mutation (HPDE-E6E7) [83]. This model aimed to characterize the early PDAC stages via growing the HPDE-E6E7 in a 3D condition by using Matrigel® as a stimulus. In the currently studied HS/ML system, it was previously reported that A818–6 HS form duct-like tube when grown in Matrigel® [27, 29]. A third model was aimed as a drug screening system for pancreatic cancer. In this model, a crowding agent (20% methyl cellulose) was used to incite the 3D aggregates in several PDAC cell lines [84]. Another PDAC in vitro 3D model focused on establishing a model that closely mimics PDAC stromal microenvironment [85]. Here, methyl cellulose was used to generate compact spheroids that entailed both PDAC cells and pancreatic stellate cells that in turn produced extensive stroma (Collagen I and III, fibronectin and smooth muscle actin). Another model that was also dedicated to create a 3D PDAC model with regard to PDAC desmoplasia, widened the scope to include a ECM submerged and air-liquid model to investigate the PDAC-stroma crosstalk during the invasion process [86]. In contrast to all these systems, our HS/ML model depends on the prevention of adhesion to elicit the 3D HS formation and it is a suitable model to investigate the involvement of the PDAC-relevant transportome in the malignant transformation.

## Conclusion

Our previous and current studies revealed a close association between the transportome and PDAC progress. The proposed quasi-normal/malignant HS/ML model of the A818–6 cells offers an unlimited transitional switching without the need of any external stimulus. We found some differentially expressed PDAC-relevant transportome genes in tumour epithelium and ML in correlation to normal pancreatic epithelium and HS. Additionally, this model emphasises the importance of cellular adhesion in the augmentation of the malignant transformation in PDAC. Altogether, our proposed HS/ML model provides a unique opportunity to study the molecular basis of the involvement of these and possibly other transportome genes, which may be regulated on the translational and/or functional level, in the malignant transformation of PDAC.

## Supplementary information

**Additional file 1: Table S1.** nCounter full transportome gene list. **Table S2.** List of the transportome genes according to IUPHAR-DB. **Table S3.** Transportome expression level in pancreatic tumour epithelium (TE) compared to normal epithelium (NE) from tissue microarray. **Table S4.** All differentially regulated genes in HS/ML cells according to the cell microarray. **Table S5.** PED enrichment of upregulated genes in Hollow spheres (HS) from the cell microarray. **Table S6.** PED enrichment of upregulated genes in monolayer (ML) from the cell microarray. **Table S7.** KEGG enrichment of upregulated HS genes in the cell microarray according to WebGestalt. Hollow sphere (HS) upregulated genes (WebGestalt), Gene Set Enrichment Analysis (GSEA) > Kyoto Encyclopedia of Genes and Genomes (KEGG) or Reactome. **Table S8.** KEGG enrichment of upregulated ML genes in the cell microarray according to WebGestalt. Monolayer (ML) upregulated genes (WebGestalt) Gene Set Enrichment Analysis (GSEA) > Kyoto Encyclopedia of Genes and Genomes (KEGG) or Reactome. **Table S9.** List of the differentially regulated transportome genes in the cell microarray.

## Data Availability

Most of the data generated or analysed during this study are included in this published article [and its supplementary information files]. Other data are available in the previously published papers [31, 32].

## Abbreviations

3D: 3 dimensional
ADM: Acinar to Ductal metaplasia
EMT: Epithelial-to-Mesenchymal transition
GO: Gene Ontology
HS: Hollow Spheres
MET: Mesenchymal-to-Epithelial transition
ML: Monolayer
PDAC: Pancreatic Ductal Adenocarcinoma
